# Modulating mitochondrial dynamics in CMT2A: a multifaceted platform for drug discovery and evaluation

**DOI:** 10.52601/bpr.2024.240037

**Published:** 2025-06-30

**Authors:** Yang Liu, Chen Yan, Borui Cao, Dejun Kong, Jiaqi Li, Wenlei Li, Yingjie Guo, Zhongyang Yuan, Yumiao Gao, Yubo Zhang, Ran Sui, Guo Chen, Xiaojiang Hao, Quan Chen

**Affiliations:** 1 State Key Laboratory of Medicinal Chemical Biology, College of Life Sciences, Nankai University, Tianjin 300071, China; 2 State Key Laboratory of Phytochemistry and Plant Resources, Kunming Institute of Botany, Chinese Academy of Sciences, Kunming 650201, China; 3 College of Pharmacy, Nankai University, Tianjin 300071, China; 4 Arc Institute, Palo Alto, CA 94304, USA; 5 Medical School, Nankai University, Tianjin 300071, China; 6 Southwest United Graduate School, Kunming 650092, China

**Keywords:** Mitofusin-2 (MFN2), Charcot-Marie-Tooth disease type 2A (CMT2A), Mitochondrial fusion, Small molecule compounds, Screening and evaluation platform, CMT2A neuronal system

## Abstract

Mitochondrial dynamics, encompassing fusion and fission processes, plays a crucial role in regulating mitochondrial distribution, motility, and material exchange within cells, particularly in the nervous system. Mitofusin-2 (MFN2), a GTPase localized to the outer mitochondrial membrane, mediates mitochondrial fusion through dimerization and conformational changes. Mutations in MFN2 are causal for Charcot-Marie-Tooth disease type 2A (CMT2A), an inherited peripheral neuropathy for which no curative treatment currently exists. Herein, we have developed a comprehensive mitochondrial drug-screening and evaluation platform to facilitate the identification of potential therapeutic candidates. This work builds upon our previous research with S89, a small molecule agonist derived from spiramine alkaloids that promotes mitochondrial fusion by interacting with endogenous MFN1 and effectively mitigates axonal degeneration in CMT2A patient-derived motor neurons. This platform integrates three sequential stages of assessment: (1) initial screening in Mfn knockout mouse embryonic fibroblasts (MEFs) to identify compounds capable of reversibly rescuing mitochondrial fragmentation; (2) evaluation in primary neuronal cultures derived from CMT2A mouse dorsal root ganglia and cortex to assess the compounds' efficacy in restoring mitochondrial morphology, axonal transport, and neurite outgrowth; and (3) final assessment in CMT2A patient-derived induced pluripotent stem cell (iPSC)-differentiated motor neurons to determine the candidates' therapeutic potential in human peripheral nervous system cells. This multi-tiered approach facilitates rapid compound screening with increasing physiological relevance, enhancing the efficiency and translational potential of identifying therapeutic candidates for CMT2A.

## INTRODUCTION

Charcot-Marie-Tooth disease type 2A (CMT2A) is a severe early-onset subtype of CMT, a spectrum of inherited neuropathies affecting 1 in 2500 individuals globally (Barreto *et al.* 2016; Feely *et al.* 2011). The disease manifests primarily as length-dependent peripheral axon degeneration, causing progressive distal muscle atrophy and sensorimotor deficits (McCray and Scherer 2021; Stuppia *et al.* 2015). Additionally, CMT2A exhibits diverse central nervous system symptoms, including early-onset stroke, antenatal encephalopathy, intervertebral disc degeneration, sensorineural hearing loss and optic atrophy (Chung *et al.* 2008; Chevrollier *et al.* 2024; Chen *et al.* 2020; Sharma *et al.* 2021), highlighting the complex genotype-phenotype relationships in this disorder.

CMT2A is primarily caused by mutations in Mitofusin-2 (MFN2), an outer mitochondrial membrane (OMM) GTPase governing mitochondrial dynamics (Giacomello *et al.* 2020; Pipis *et al.* 2020; Zuchner *et al.* 2004). MFN2 mediates mitochondrial fusion through GTP-dependent dimerization and conformational change (Li *et al.* 2019; Legros *et al.* 2002), while regulating mitochondrial transport along microtubules by interacting with the Miro/Milton complex (Misko *et al.* 2010). These coordinated processes facilitate mitochondrial content exchange, repair, and energy distribution. Disease-causing mutations in MFN2 disrupt these functions, resulting in mitochondrial fragmentation, ultrastructural defects, reduced mitochondrial DNA (mtDNA) content and compromised energy production (Westermann 2010). These mitochondrial defects particularly affect neurons with high metabolic demands at synapses and lead to widespread pathological changes in innervated tissues.

The Mendelian inheritance pattern and predominant familial occurrence of CMT2A are attributed to mutations in the nuclear gene encoding MFN2 (Braathen 2012; Chung *et al.* 2006; Verhoeven *et al.* 2006). To date, over 100 dominant nonsynonymous CMT2A mutations have been identified in MFN2 (Ferre 2024). Recent crystallographic studies of truncated human MFN2 by Li *et al*. have elucidated the molecular dynamics of mitochondrial membrane fusion, revealing that the sustained dimers formed by MFN2 post-GTP hydrolysis are crucial for efficient membrane tethering (Li *et al.* 2019). Their investigation of several CMT2A-causing mutations demonstrated variable effects on GTPase activities and MFN2 conformational changes, underscoring the molecular complexity underlying CMT2A pathogenesis.

The complexity of CMT2A stems from the intricate interplay between mitochondrial dysfunction and nuclear genetic inheritance. Recent advances in understanding MFN conformational transitions during mitochondrial fusion have provided insights into how MFN2 point mutations perturb these dynamic processes, contributing to CMT2A pathology. Higher eukaryotes express two mitofusins, MFN1 and MFN2, each comprising a G domain for GTP hydrolysis, two helix bundle domains (HB1 and HB2), and two transmembrane domains (Huang *et al.* 2017; Ishihara *et al.* 2004). Their cytosol-facing carboxy and amino termini facilitate OMM tethering through the formation of homodimers (MFN1-MFN1, MFN2-MFN2) or heterodimers (MFN1-MFN2) (Detmer and Chan 2007; Dorn 2019).

In the basal state, MFN is auto-inhibited by G domain binding to HB2. GTP recruitment induces G domain detachment from HB2, resulting in an “unlocked” MFN conformation. Subsequent GTP hydrolysis triggers inward movement of HB1, facilitating OMM apposition and fusion (Cao *et al.* 2017; Huang *et al.* 2017). This OMM fusion process is a prerequisite for the complete fusion of two mitochondria. One of the most prevalent CMT2A-associated mutations occurs at MFN2 amino acid residue 94 (R94W or R94Q), located within the hinge region upstream of the G domain. These mutations are hypothesized to disrupt MFN2-MFN2 dimerization and perturb MFN2 conformational transitions (Li *et al.* 2019), thereby impacting the dynamic processes of mitochondrial fusion.

In our recent work, Guo *et al*. generated a small molecule library derived from natural compound spiramines (Hao *et al.* 1987) and identified S89 as capable of rescuing mitochondrial fragmentation in Mfn2 knockout mouse embryonic fibroblasts (Mfn2 KO MEFs) in the presence of Mfn1 (Guo *et al.* 2023). S89 directly interacts with endogenous MFN1 at a specific loop region within the HB2 domain, competitively disrupting its interaction with the G domain. This interaction releases MFN1 from its auto-inhibited state, enhancing its GTPase activity, G domain dimerization, and subsequent membrane fusion capability. Notably, evaluation in motor neurons derived from induced pluripotent stem cells (iPSCs) of CMT2A patients demonstrated S89’s ability to rescue aberrant mitochondrial ultrastructural morphology, restore mitochondrial membrane potential homeostasis, and promote neurite outgrowth. Furthermore, S89 alleviated mitochondrial defects caused by heart ischemia/reperfusion injury in mouse models (Guo *et al.* 2023).

Based on these findings, S89 emerges as a promising candidate for the treatment of MFN2 mutation-induced neurodegenerative disease CMT2A. Building on this research, we have generated a compound library derived from spiramines, including analogues of S89, some of which exhibit improved synthesis efficiency and yield (unpublished data). We speculate that screening this library may identify additional candidates capable of rescuing mitochondrial fragmentation or neurodegeneration. Given the time- and resource-intensive nature of obtaining differentiated motor neurons from CMT2A patients, accelerating the efficiency of early-stage drug screening and evaluation necessitates the establishment of a systematic screening platform.

**Figure 1:**
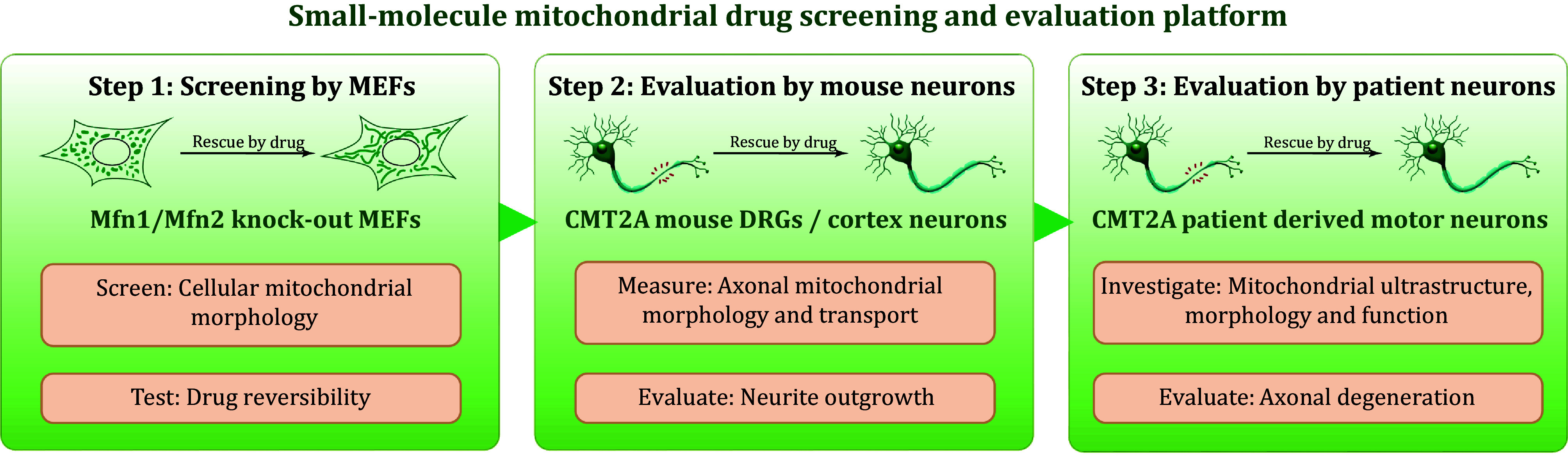
Schematic diagram of the multi-tiered mitochondrial drug screening and evaluation platform for CMT2A therapeutics. The platform is based on Mitofusins knockout mouse embryonic fibroblasts (Mfn KO MEFs), primary neuronal cultures from CMT2A mouse dorsal root ganglia (DRGs) and cortex, and CMT2A patient-derived motor neurons. Indicators for assessing candidate compounds are listed in orange boxes

Herein, we describe a multi-tiered drug-screening and evaluation platform for identifying potential therapeutic compounds for CMT2A, by integrating Mfn KO MEFs, primary neuronal cultures from CMT2A mouse models, and patient-derived neuronal cultures (Fig. 1). Briefly, we aim to initially identify candidate compounds capable of reversibly rescuing mitochondrial fragmentation observed in Mfn2 KO cells. These candidates undergo evaluation in primary neuronal cultures from CMT2A mouse models, focusing on mitochondrial morphology, transport, and neurite outgrowth. Finally, promising molecules are assessed in CMT2A patient-derived iPSC-differentiated motor neurons to determine their potential for application to the human peripheral nervous system.

## RESULTS

### Preliminary screening using Mitofusins knock-out cells

**Figure 2:**
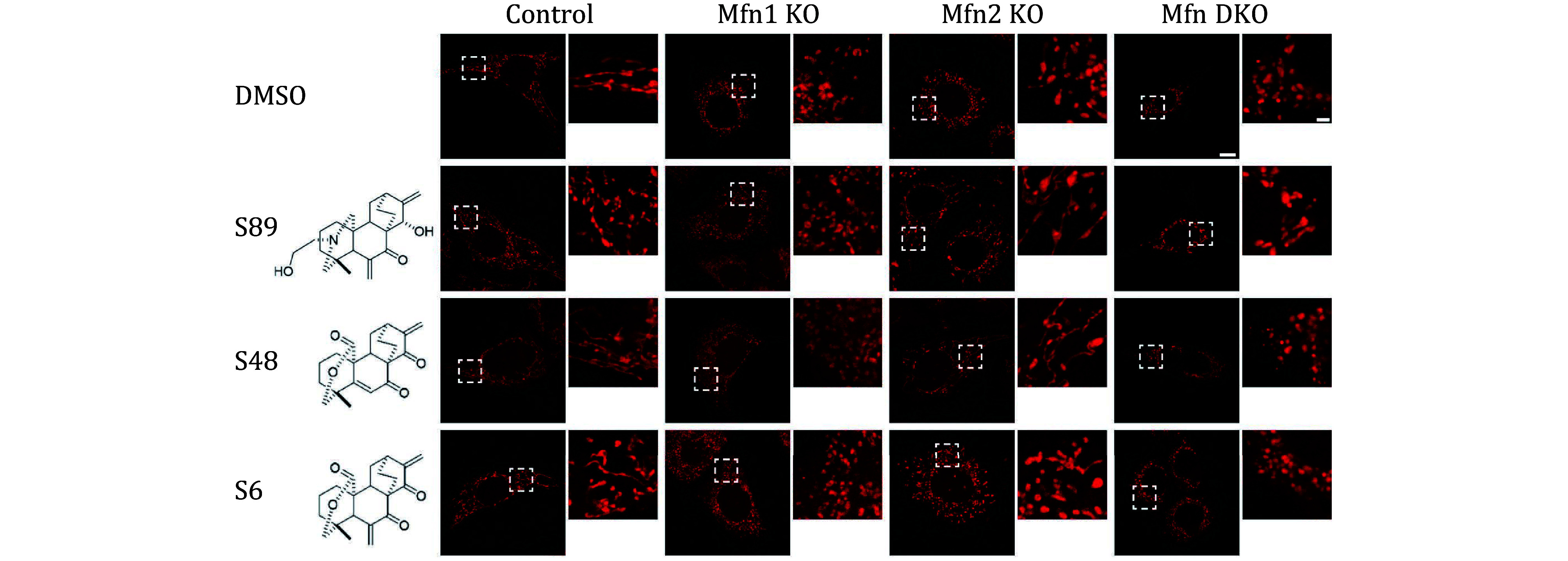
Preliminary screening of compounds for mitochondrial fragmentation rescue. Representative confocal images showing mitochondria labeled with MitoTracker Red in wild type (Control), *Mfn1* knock-out (Mfn1 KO), Mfn2 KO and *Mfn1*/*2* double KO (Mfn DKO) MEF cells after treatment with DMSO or 2 μmol/L compounds for 24 h. Each cell line is represented by an overview image (Left panels, scale bar, 10 μm) and a magnified image of the outlined mitochondrial region (Right panels, scale bar, 2 μm)

Mouse embryonic fibroblasts (MEFs) deficient in either *Mfn1* (Mfn1 KO), *Mfn2* (Mfn2 KO), or both genes (*Mfn* double knockout, Mfn DKO) exhibit fragmented mitochondria due to impaired fusion events. Given that CMT2A is caused by mutations in MFN2, not MFN1, we hypothesized that compounds capable of rescuing mitochondrial fragmentation in Mfn2 KO MEFs may have therapeutic potential for CMT2A patients. As illustrated in Fig. 2, S89 effectively mitigated mitochondrial fragmentation in Mfn2 KO MEFs, with no discernible effects observed in Mfn1 KO or Mfn DKO MEFs. This observation suggests S89's specificity in ameliorating Mfn2 deficits in the presence of Mfn1. In this initial screening phase, candidate molecules were screened using Mfn KO MEFs, with priority given to compounds capable of inducing mitochondrial elongation. Regarding the efficacy of rescuing mitochondrial fragmentation, a representative compound S48 demonstrated comparable potency to S89, while S6 exhibited lower efficiency (Fig. 2).

**Figure 3:**
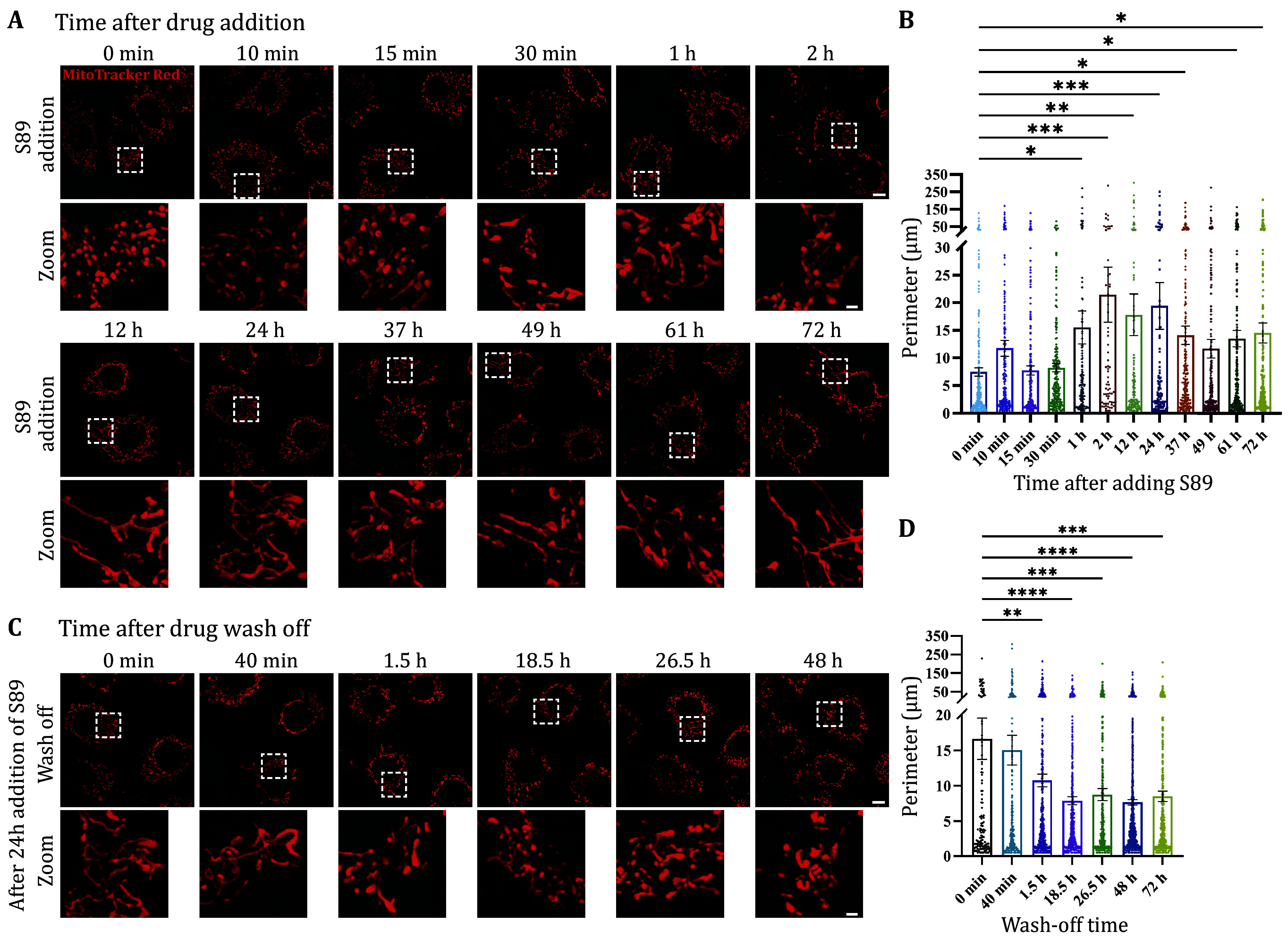
Reversibility test of small-molecule candidates in altering mitochondrial morphology. **A**,**B** Evaluation of onset time. Live Mfn2 KO MEFs stained with MitoTracker Red and treated with 2 μmol/L S89 for indicated time points. **C**,**D** Duration of efficacy after drug wash-off. Mfn2 KO MEFs were treated with 2 μmol/L S89 for 24 h, stained with MitoTracker Red, then subjected to drug wash-off. **A**,**C** Representative confocal images. Top panels, overview images (Scale bar, 10 μm). Bottom panels, magnified images of mitochondrial morphology (Scale bar, 2 μm). **B**,**D** Quantification of mitochondrial perimeters. Data are represented as mean ± SEM. Dunnett’s ANOVA test was used for multiple comparisons. *p < 0.05, **p < 0.01, ***p < 0.001, ****p < 0.0001

To mitigate potential off-target effects and adverse reactions, candidate compounds are evaluated for reversibility through add-in and wash-off experiments. Compounds forming covalent bonds with endogenous proteins, as evidenced by irreversible effects in these experiments, are excluded from further consideration. To quantitatively assess the efficacy of small-molecule drugs in add-in experiments, we defined the Half Fusion Time (FT50) as the duration of drug treatment at a specific concentration required for 50% of Mfn2 KO cells to exhibit mitochondrial fusion. Upon addition of 2 μmol/L S89, 50% of Mfn2 KO MEFs displayed mitochondrial elongation within 10 min, yielding an FT50 (2 μmol/L, S89) of 10 min (Fig. 3A). Prolonged S89 treatment for one hour resulted in significant mitochondrial elongation (Figs. 3A and 3B). Importantly, the rescue effect was reversible, with mitochondrial morphology returning to baseline after S89 washout for longer than 1.5 h (Figs. 3C and 3D). These results suggest that S89 is a promising candidate capable of reversibly ameliorating mitochondrial fragmentation.

Subsequently, molecules demonstrating long-lasting reversibility underwent further pharmacological testing, including assessment of cellular uptake and decay rates, determination of maximal effective concentration, and evaluation of cell viability (Guo *et al.* 2023). Compounds that efficiently and reversibly induce mitochondrial fusion, exhibit suitable cellular uptake and decay kinetics, and demonstrate low toxicity were selected as valuable candidates for evaluation in neuronal cultures derived from CMT2A animal models and patient samples.

### Evaluation by CMT2A mouse neurons

**Figure 4:**
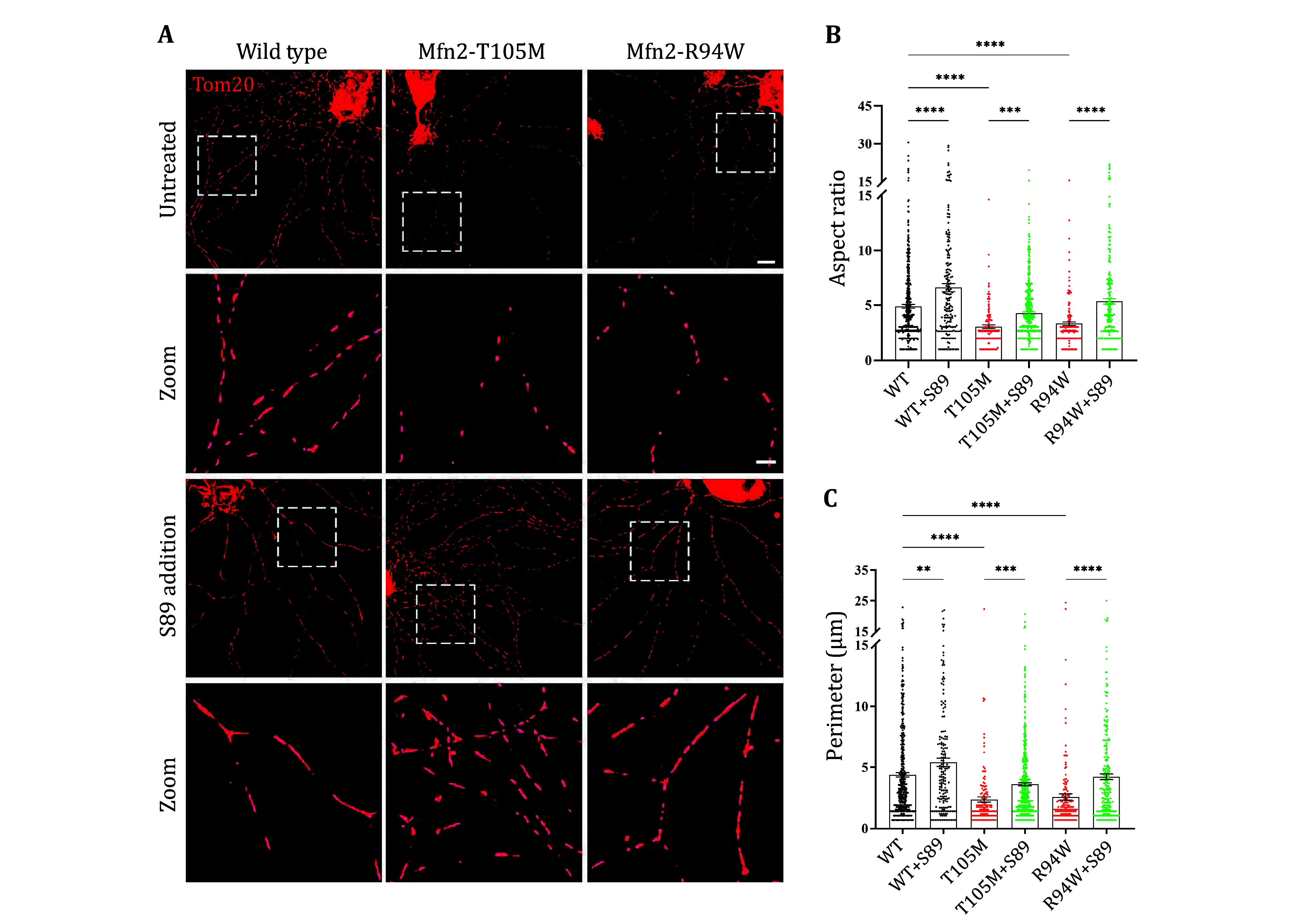
Evaluation of S89 efficacy in CMT2A mouse dorsal root ganglion (DRG) neurons. **A** Representative confocal images of mitochondria immunostained with anti-Tom20 in 3-week-old wild-type (WT), Mfn2-T105M and Mfn2-R94W mouse DRG neurons, with and without 2 μmol/L S89 treatment for 12 h. Scale bar in overview images: 15 μm. Scale bar in magnified images: 5 μm. **B**,**C** Quantification of mitochondrial aspect ratios and perimeters in DRG axons. Data are represented as mean ± SEM. Brown-Forsythe and Bartlett’s ANOVA tests were used for multiple comparisons. **p < 0.01, ***p < 0.001, ****p < 0.0001

Dorsal root ganglia (DRGs) are clusters of sensory neuronal cell bodies located at the dorsal root of the spinal cord. Each DRG neuron projects a single axon that bifurcates into peripheral and central branches, facilitating the transmission of sensory information from peripheral tissues to the central nervous system (Nascimento *et al.* 2018). As CMT2A is primarily characterized by peripheral nerve degeneration, we established an *in vitro* mouse DRG culture system to investigate axonal mitochondrial morphology and assess drug efficacy. Using CRISPR/Cas9-mediated genome editing, we generated two CMT2A knock-in mouse models, each carrying a distinct Mfn2 point mutation: T105M or R94W. The R94W mutation, the most prevalent in CMT2A populations, affects MFN2 conformational changes during GTP hydrolysis, while T105M impairs G interface and disrupts MFN dimer formation (Li *et al.* 2019). DRGs were dissected from 3-week-old CMT2A mice and cultured for two days. As anticipated, axonal mitochondria in both T105M and R94W DRG cultures exhibited fragmentation, and neurite outgrowth was reduced compared to wild-type controls (Fig. 4). After treatment with S89, both the mitochondrial fragmentation and impaired neurite outgrowth were corrected to levels comparable to those observed in normal wild-type neurons (Fig. 4).

**Figure 5:**
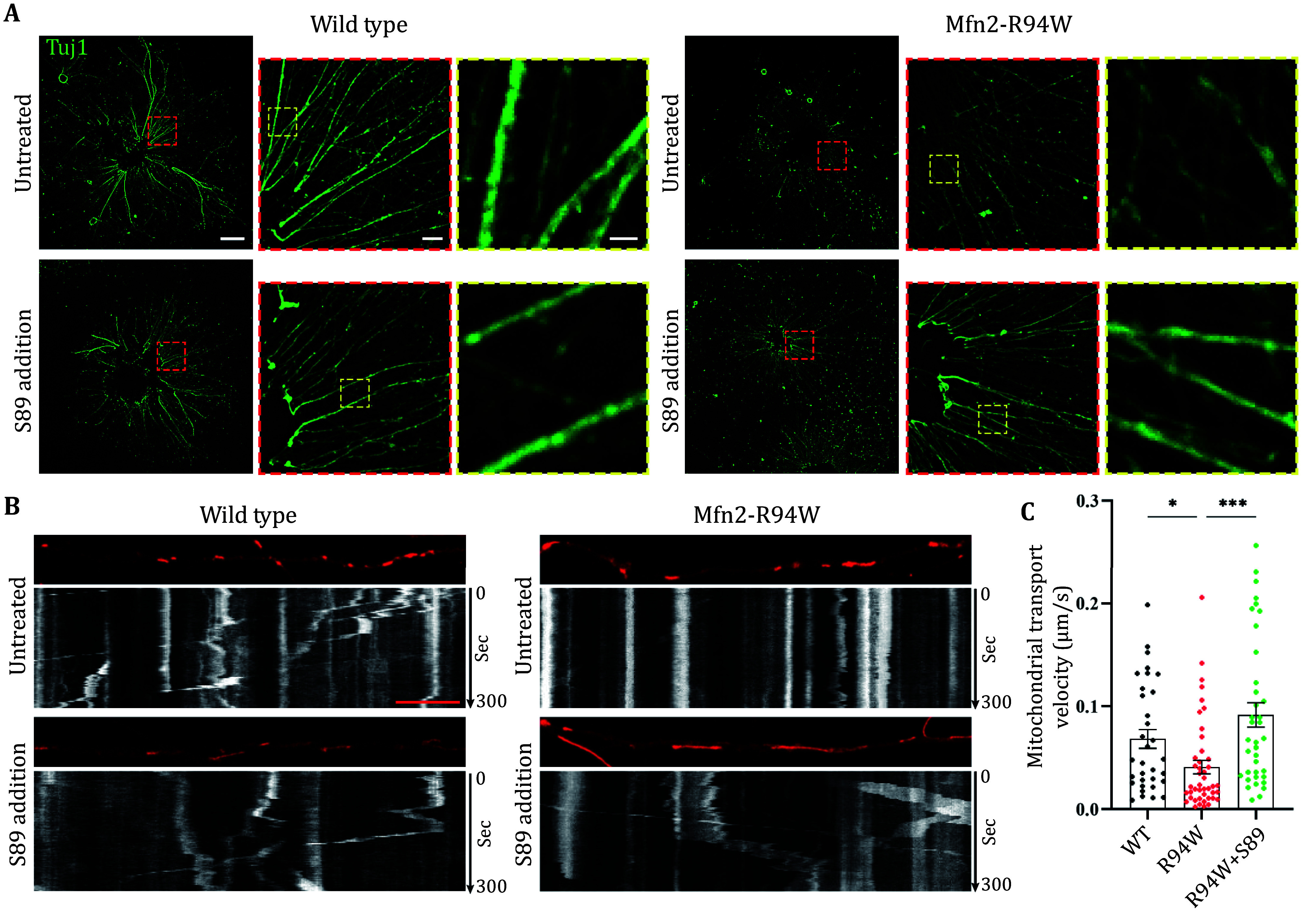
Evaluation of S89 in mouse cortex *ex vivo* culture system. **A** Immunostaining of Tuj1 in mouse cortex *ex vivo* cultures from wild-type (WT) and Mfn2 R94W mutant neonatal mice, with or without 2 μmol/L S89 treatment for 3 d. For each genotype: left panels show whole view images of cortex explants (Scale bar: 400 μm); middle panels show magnified images of spreading neurites outlined in red (Scale bar: 50 μm); right panels show magnified images of axonal morphologies outlined in yellow (Scale bar, 10 μm). **B** Representative kymographs of mitochondrial transport after treatment with 2 μmol/L S89 for 3 d or without treatment. Mitochondria are labeled by live-cell mitochondrial probe PK MitoTracker Orange (20 nmol/L). Mitochondrial transport was observed for 300 s. Scale bar: 10 μm. **C** Average mitochondrial transport velocities during the imaging period. Data are represented as mean ± SEM. Brown-Forsythe and Welch ANOVA tests were used for multiple comparisons. *p < 0.05, ***p < 0.001

CMT2A also affects the central nervous system (CNS). A recent psychiatric survey of Italian CMT patients revealed higher levels of anxiety, depression, and general distress compared to control groups (Bellofatto *et al.* 2023). Similar affective symptoms have been observed in our CMT2A mouse models (unpublished data). We hypothesized that CNS development is impaired due to CMT2A-related Mfn2 mutations. To investigate this hypothesis, we employed a neonatal (P0) mouse cortex *ex vivo* culture system to visualize and quantify mitochondrial movement and neuronal development. Cortical blocks were seeded in culture media, with neurite outgrowth visible after three days (Fig. 5A). This ‘dissect-and-seed’ *ex vivo* culture approach circumvents mechanical and enzymatic dissociation, preserving neuron-to-glia ratios and cell–cell interactions during seeding, thereby enhancing cortical neuronal survival. Moreover, initiating the *ex vivo* culture at the neonatal stage, when the majority of cortical neurogenesis is complete, expedites the drug evaluation process at the neuronal culture level.

Axonal mitochondria were labeled using the live-cell mitochondrial probe PK MitoTracker Orange (Liu *et al.* 2022). Mitochondrial transport was recorded through live-cell imaging and visualized as kymographs (Fig. 5B). Compared to wild-type cortical neurons, Mfn2 R94W mutants exhibited reduced axonal mitochondrial transport velocities (Fig. 5C). Additionally, mutant cortical neurons displayed axonopathy, characterized by axonal shrinkage, aberrant excessive branching, and axonal thinning (Fig. 5A). These abnormalities were ameliorated by the addition of S89, suggesting its potential efficacy in further investigations using CMT2A animal models or patient-derived neuronal cultural systems.

### Evaluation using CMT2A patient-derived neurons

Motor neurons differentiated from induced pluripotent stem cells (iPSCs) of CMT2A patients provide a valuable *in vitro* model for studying the human peripheral nervous system, enabling drug evaluation and mitochondrial characterization specific to CMT2A pathology. In our evaluation platform, peripheral blood mononuclear cells (PBMCs) were isolated from two CMT2A patients carrying MFN2 R94W and T105M mutations, respectively. These PBMCs were reprogrammed into iPSCs and subsequently differentiated into motor neurons. The resulting mutant neurons exhibited several pathological features, including pronounced axonal atrophy, mitochondrial membrane potential depolarization, axonal mitochondrial fragmentation, and aberrant mitochondrial cristae structure (Guo *et al.* 2023). Following treatment with S89, the axonopathy was effectively mitigated, resulting in a restoration of axonal morphology and mitochondrial structure to a state resembling that of healthy control neurons (Guo *et al.* 2023).

## DISCUSSION

### Role of drug development in elucidating the mechanisms underlying CMT2A

In the field of neurodegenerative diseases, there exists a synergistic relationship between mechanism elucidation and drug development. Genetic model-based research illuminates critical nodes in disease pathogenesis, revealing potential therapeutic targets. Conversely, the observed therapeutic outcomes following drug administration provide insights into drug-target interactions, binding affinities, and functional consequences (Swinney and Anthony 2011), thereby advancing our understanding of the molecular mechanisms underlying diseases.

Given its frequent association with MFN2 mutations, CMT2A research has emphasized the pivotal role of MFN2 conformational plasticity and mitochondrial dynamics in drug design. S89, a spiramine derivative, has demonstrated efficacy in ameliorating mitochondrial fragmentation in Mfn2 KO MEFs, CMT2A patient-derived motor neurons, and mouse hearts subjected to ischemia/reperfusion injury (Guo *et al.* 2023). TAT–MP1^Gly^, a cell-permeant minipeptide, has shown the ability to rectify mitochondrial abnormalities in mouse fibroblasts and neurons expressing human MFN2 transgenic mutations (Franco *et al.* 2016; Zhou *et al.* 2019).

These studies not only present potential therapeutic agents for CMT2A but also collectively emphasize the essential role of MFN1 in effective treatment. Human MFN1 exhibits faster GTP turnover and GDP off-rates compared to MFN2, rendering MFN1 less efficient at maintaining the “unlocked” conformation and the sustained dimers necessary for fusion post-GTP hydrolysis (Li *et al.* 2019). However, MFN1 and MFN2 exhibit complementary functions in mediating mitochondrial tethering and fusion, providing valuable insights for the design of compounds capable of manipulating MFN1 conformations to treat CMT2A caused by MFN2 mutations (Detmer and Chan 2007; Ishihara *et al.* 2004). Mechanistically, S89 competitively binds to the HB2 loop region of MFN1, disrupting the HB2-G domain intramolecular interaction in the auto-inhibited state (Guo *et al.* 2023). This process induces HB2-G detachment in MFN1, mimicking the activated state achieved through GTP binding (Cao *et al.* 2017). Consequently, S89 promotes the conformational transition required for MFN1-mediated OMM tethering and fusion. This research provides the first direct evidence of a specific therapeutic target in CMT2A treatment beyond MFN2 itself (Guo *et al.* 2023).

Our study utilized a comprehensive mitochondrial drug screening and evaluation platform to assess S89's efficacy across multiple model systems, including Mfn2 KO MEFs, cultured CMT2A mouse neurons and CMT2A patient-derived motor neurons. The results validate the robustness of this platform in efficiently identifying promising therapeutic candidates for CMT2A treatment and potentially uncovering additional targets for further elucidation of disease pathogenesis.

### Potential of CMT2A mouse models in mitochondrial drug assessment and therapeutic rescue

It is noteworthy that endogenous CMT2A-related Mfn2 mutant mice do not exhibit significant peripheral neurodegenerations under standard housing conditions, contrasting with the symptomatic presentation in human patients. Considering the potential role of environmental stress in exacerbating neurodegeneration, Cui *et al*. investigated multiple subtypes of CMT2 mouse models under high-intensity exercise conditions, proposing a stress granule (SG)-related mechanism. Their findings suggest that aberrant protein interactions in CMT2 SGs disturb the SG network and stress responses, rendering motor neurons more vulnerable to environmental stressors, and potentially contributing to disease pathogenesis (Cui *et al.* 2023). Given the recognized influence of environmental risk factors in precipitating neurodegeneration, and considering that other CMT mutant mouse models have demonstrated neuromuscular junction and behavioral deficits under stress, it is imperative to subject Mfn2 mutant mice to various stress conditions in future studies. This exploration aims to determine whether such stressors can augment the susceptibility of mutant mice to neurodegeneration, potentially bridging the gap between mouse models and human pathology.

Neuronal culture systems are widely employed tools for mechanism dissection and drug evaluation in neurodevelopment and neurodegeneration research. Based on the CMT2A Mfn2 point mutant mouse model, our study utilized dorsal root ganglion (DRG) *in vitro* and cortex *ex vivo* systems to assess potential compounds for rescuing mitochondrial morphology and axonal transport deficits. Previous studies have indicated that DRGs from Mfn2 R94W point mutant embryos do not exhibit significant reductions in mitochondrial transport velocity or elevated levels of mitochondrial fragmentation compared to wild type (Strickland *et al.* 2014). In our experimental setup, we utilized DRGs from 3-week-old mutant mice and observed defects in mitochondrial morphology and neurite outgrowth, both of which were corrected by treatment with S89. This suggests that peripheral neurodegeneration in CMT2A may be dependent on the developmental stage, underscoring the suitability of our DRG culture system for evaluating mitochondria-targeted compounds.

A notable feature of our screening and evaluation platform is the inclusion of the cortex *ex vivo* culture step. In addition to peripheral nervous system (PNS) involvement, central nervous system (CNS) manifestations in CMT2A patients are increasingly reported, including early onset stroke, hearing loss, and optic atrophy. To investigate CNS involvement, we dissected cortical tissue from P0 neonatal mice, a developmental stage when the majority of neurogenesis is complete. Our aim is to detect neurodevelopmental defects at this stage and establish correlations with CNS symptoms observed in patients.

The cortical explants, which preserve developed axons and intrinsic synaptic connections, rapidly extend axons on culture plates, facilitating the observation of neurite morphology and mitochondrial dynamics within a short timeframe of three days. As hypothesized, we observed developmental deficits in Mfn2-R94W cortical neurons, manifested by impaired mitochondrial movements and neurite outgrowth. Notably, these phenotypes were rescued by S89 treatment. This “dissect-and-seed” (DNS) method not only provides a convenient approach for evaluating mitochondria-targeted compounds but also addresses the gap in research on CNS-related mechanisms in CMT2A. The CMT2A mouse model may present additional CNS symptoms, offering insights for comprehensive pathogenic investigations and potentially expanding our understanding of the disease beyond its classical peripheral neuropathy presentation.

### Comparative analyses between CMT2A patients and animal models

Adult CMT2A mutant mice exhibit peripheral neuronal deficits that are less severe compared to those observed in human patients. One possible explanation for this discrepancy lies in the amino acid variance of MFN1 between mice and humans. In the human MFN1 protein, the conserved amino acid residue responsible for GTP binding and hydrolysis has evolved from Thr108 to Ile108, in contrast to its homolog MFN2 Thr129 (Li *et al*. 2019, Fig. 6). This evolutionary change in human MFN1 results in an increased GTP turnover rate but a decreased ability to form dimers through *trans-*association. In CMT2A patients with MFN2 mutations, MFN1 is unable to fully compensate for the loss of MFN2's dimer formation function, thereby potentially contributing to CMT2A pathogenesis. However, in mice, this residue remains as threonine in both Mfn1 and Mfn2. Consequently, even in the presence of an Mfn2 mutation, murine Mfn1 maintains a *trans* association as robust as that of intact Mfn2, which may mitigate the severity of symptoms in mouse models.

**Figure 6:**
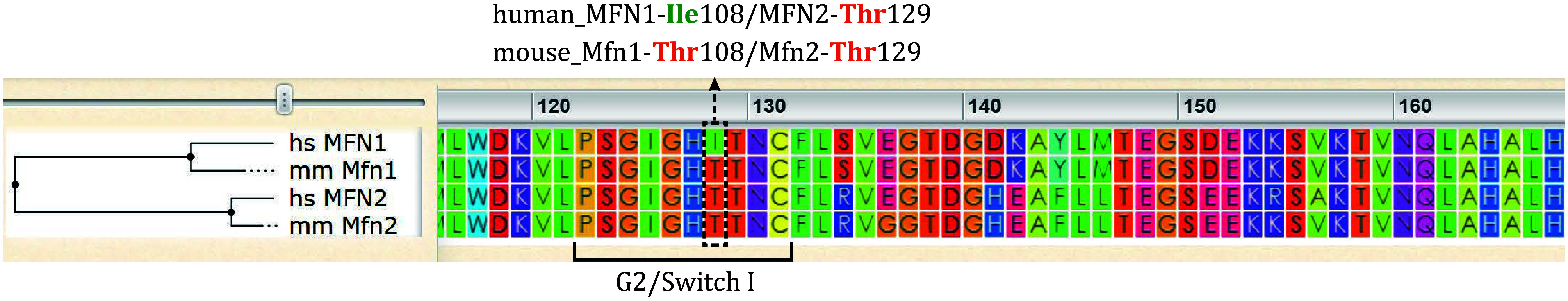
Sequence alignment of the G2/Switch I region of mitofusins from human and mouse. The dashed box highlights the evolved residue human MFN1-Ile108, compared to mouse Mfn1-Thr108 and mouse/human MFN2-Thr129

Another potential explanation for the phenotypic disparity between humans and mice in CMT2A pathology is the difference in anatomical scale, particularly in limb size. Human peripheral neuronal axons are substantially longer compared to those in mice. Given that MFN2 mutations have been reported to impair mitochondrial axonal transport, this may exacerbate the challenge of transporting mitochondria to human axon terminals, including neuromuscular junctions, for energy support. This difference in mitochondrial transport dynamics likely contributes to the observed phenotypic variation between humans and mice in CMT2A pathology.

Interspecies variations in mitofusin protein sequences and neurological structures potentially explain the discrepancy in CMT2A pathogenesis between humans and mice, raising questions about the clinical relevance of our mouse model-based drug screening pipeline. Our initial screening employs Mfn-knockout MEFs, where mitochondrial fusion depends on all three dimer configurations: Mfn1-Mfn1, Mfn2-Mfn2, and Mfn1-Mfn2 (Chen *et al.* 2003). Thus, the knockout of either Mfn1 or Mfn2 leads to mitochondrial fragmentation. This cell-type-specific dependency on both Mfn1 and Mfn2 makes MEFs particularly suitable for initial drug screening, in contrast to cells like trophoblast giant cells that primarily rely on Mfn2-Mfn2 dimers for mitochondrial fusion. Additionally, the rationale for the initial use of MEFs was their ease of generation from Mfn-knockout mice and their ability to be immortalized, providing a cost-effective and tractable system for high-throughput screening.

To enhance clinical relevance, we validated candidate drugs using human cell lines. We generated MFN1 and MFN2 knockout HeLa cells via CRISPR/Cas9 that exhibit mitochondrial fragmentation. S89 rescued this phenotype in MFN2-knockout HeLa cells expressing MFN1, consistent with MEF results (Guo *et al.* 2023). Despite species-specific differences in MFN2 mutation effects, both mouse and human cell models showed concordant responses to S89 treatment. Furthermore, motor neurons differentiated from CMT2A patient-derived iPSCs showed improved mitochondrial morphology and neurite outgrowth upon S89 treatment, supporting its therapeutic potential in human disease.

The differential impact of limb length on axonal mitochondrial transport between humans and mice represents a critical physiological variable that cannot be fully recapitulated *in vitro* due to spatial constraints of neuronal cultures. Nevertheless, cultured mouse cortical neurons and CMT2A patient-derived neurons carrying MFN2 mutations exhibit mitochondrial transport deficiencies and axonal atrophy (Fig. 5, Guo *et al.* 2023), which partially model the energy distribution challenges in human long axons. Thus, drugs that rescue these phenotypes may still effectively target CMT2A pathology. Therefore, the mouse neuronal culture system serves as a cost-effective and mechanistically informative evaluation platform within our screening pipeline.

Comparative analyses between CMT2A patients and animal models are essential to advance our understanding of the disease. Bioinformatic analyses focusing on differences in Mfn2 or mitochondria-regulated networks may reveal crucial genetic nodes underlying CMT2A pathogenesis. Additionally, the establishment of CMT2A animal models more closely related to humans, such as non-human primates, offers valuable insights into disease mechanisms and enhances therapeutic development. These approaches collectively contribute to a more comprehensive understanding of CMT2A pathophysiology and may ultimately lead to more effective translational strategies for therapeutic interventions.

## MATERIALS AND METHODS

Primary and secondary antibodies used in this work are listed in Table 1, and reagents, chemicals and medium are listed in Table 2.

**Table 1:** Primary and secondary antibodies in this work

Antibodies	Source	Catalogue No.
Anti-Tuj1	ABclonal	A17913
Anti-Tom20	BD Bioscience	612278
Alexa Fluor^TM^ 488 goat	Invitrogen	A11008
Alexa Fluor^TM^ 546 goat	Invitrogen	A11038

**Table 2:** Reagents, chemicals and medium in this work

Reagents	Source	Catalogue No.
DMEM	Gibco	C11995500BT
Fetal Bovine Serum	Biological Industries	04-001-1ACS
DMSO	Merck	472301
HBSS (with Ca^2+^, Mg^2+^)	Solarbio	H1025
Live Cell Imaging Solution	Invitrogen	A14291DJ
Matrigel Matrix (Growth factor reduced)	Corning	354230
Poly-L-Ornithine	Sigma	P3655
Laminin	Roche	114956-81-9
Collagenase/Dispase	Roche	COLLDISP-RO
Neurobasal^TM^-A medium	Gibco	10888022
L-glutamine	Gibco	25030-81
B27 supplement 50X	Gibco	17504-044
Penicillin/Streptomycin, sterile (100 X, 10k U/mL Penicillin, 10 mg/mL Streptomycin)	Meilunbio	MA0110
MitoTracker™ Red CMXRos	Invitrogen	M7512
PK MitoTracker Orange	Gift from Dr. Zhixing Chen in Peking University National Biomedical Imaging Center (Liu *et al.* 2022)	PKMO-1
Paraformaldehyde	Aladdin	C104188
Triton X-100	Sigma	T8787
Albumin Bovine V (BSA)	Solarbio	A8020
Mounting Medium, antifading (with DAPI)	Solarbio	S2110

### Mouse embryonic fibroblast cell culture and preliminary screening

The Mfn1 KO, Mfn2 KO and Mfn DKO MEFs were provided by David Chan at Caltech (Chen *et al.* 2003). MEFs were cultured in MEF medium (DMEM containing 10% FBS and 1× Penicillin/Streptomycin) at 37°C in a 5% CO_2_ atmosphere. Cells were passaged every 2 to 3 d when confluency reached approximately 70% to 90%. For the preliminary screening of the compounds, MEFs were plated in 24-well plates, with a cover slip in each well. Considering the differences in growth rates, the plating density of wild type, Mfn1 KO and Mfn2 KO MEFs was 2500 cells per well, while Mfn DKO was 5000 cells per well. After 12 h, 2 µmol/L mitochondrial compounds or DMSO (vehicle control) were added to corresponding wells. Following 24 h of treatment, 20 nmol/L MitoTracker Red (fixable) was added to label mitochondria. Samples were then fixed with 4% paraformaldehyde in PBS for 20 min at 37°C, followed by three 5-min PBS washes. Finally, the samples were mounted in an antifade mounting solution and imaged using a Leica SP8 confocal microscope.

### Mouse dorsal root ganglia *in vitro* culture

The NIH Guide for the Care and Use of Laboratory Animals was followed with approval from the Committee of Animal Research at Nankai University.

The day before dorsal root ganglion (DRG) isolation, the glass bottom cell culture dishes (SOFRA Life Science) were coated with 25 µg/mL Poly-L-Ornithine in PBS at 37°C overnight, followed by three 60-min PBS washes and one ddH_2_O wash at room temperature. The dishes were then coated with 5 µg/mL Laminin in PBS at 37°C for 1 h and prepared for DRG cultures. The DRGs were dissected from the spinal cords of 3-week-old mice, with the axon bundles removed in sterile ice-cold HBSS containing Ca^2+^ and Mg^2+^. The DRGs were washed with sterile PBS once and digested in 1 mg/mL Collagenase/Dispase in PBS in a 5% CO_2_ atmosphere at 37°C for 30 min, followed by 30-min digestion at room temperature on an orbital shaker with a speed of 50 r/min. The digested DRGs were centrifuged at 1000*g* for 2 min at 4°C and resuspended with 1 mL DRG medium (DMEM with 50 ng/mL NGF, 10% horse serum, 2 mmol/L L-glutamine, 1× Penicillin/Streptomycin). After filtered by 70 µm cell strainers and counted, the DRG neurons were plated in the pre-coated dishes, with a density of 1000 cells/cm^2^. The medium was renewed after 2 h. The next day, half of the DRG medium was replaced and samples were fixed after 48 h of culture. S89 (2 µmol/L) or DMSO was added to the medium 12 h before sample collection.

### Primary mouse cortex *ex vivo* culture

P0 neonatal mice were anesthetized with isoflurane and disinfected with 75% ethanol, followed by brain cortex dissection in cold sterile HBSS containing Ca^2+^ and Mg^2+^, with meninges and blood vessels removed. The cortex regions above the hippocampi were collected and cut into small pieces less than 0.1 mm^3^. Cortex pieces were kept in ice-cold Live Cell Imaging Solution (1.5 mL per neonatal mouse) until cortex pieces from all mice were collected and ready for subsequent steps. Samples were washed twice with HBSS (containing Ca^2+^ and Mg^2+^) and plated in glass-bottom cell culture dishes (SOFRA Life Science) pre-coated with 2 % Matrigel in DMEM at 37°C overnight. The cortex pieces were cultured using NBMA medium (Neurobasal^TM^-A medium, 1× B27, 2 mmol/L L-glutamine, 1× Penicillin/Streptomycin) in a 5% CO_2_ atmosphere at 37°C. Half of the medium was replaced with fresh NBMA after 2 d of culture.

### Compound reversibility test

In the evaluation of the compound onset time, Mfn2 KO MEFs were seeded into confocal dishes containing 1.5 mL of MEF medium. Once the cells had attached to the bottom, 20 nmol/L MitoTracker Red was added to label the mitochondria. The dishes were then transferred to an SP8 confocal microscope, maintained at 37°C and 5% CO_2_ for live-cell imaging. Images taken before adding compounds were recorded at 0 min. Compounds (2 μmol/L) were then immediately added to the medium and imaging was performed at indicated time points based on the drug effects and experimental requirements.

To test the duration of efficacy, compound wash-off experiments were performed. After cells were attached to the bottom, compounds (2 μmol/L) were added to the medium, followed by a 24-h incubation. Before live-cell imaging, 20 nmol/L MitoTracker Red was added to the medium. Dishes were transferred to the SP8 confocal microscope maintained at 37°C temperature and 5% CO_2_. Cells were washed three times with Live Cell Imaging Solution containing 20 nmol/L MitoTracker Red. Imaging was then immediately performed and recorded as 0 min. Subsequently, images were then taken at indicated time points to evaluate mitochondrial morphological changes.

### Live-cell imaging of cortical neurons

Cortical explants exhibiting neurite outgrowth were stained with 20 nmol/L PK MitoTracker Orange mitochondrial probe (Genvivo Tech) at 3 d post *ex vivo* culture. Mitochondrial axonal transport was visualized in the Live Cell Imaging Solution containing PK MitoTracker Orange. Images were captured using a Leica SP8 confocal microscope. Time-lapse recordings of labeled mitochondrial movements were acquired every 3 s for 5 min.

### Immunofluorescence and microscopy

Cultured cortical explants were fixed in PBS containing 4% paraformaldehyde at 37°C for 20 min, followed by three times of 5-min PBS washes at room temperature. The samples were permeabilized with 0.2 % Triton X-100 for 10 min at 4°C, followed by incubation with 5% BSA in PBS for 1 h at room temperature. Cortical samples were then incubated with primary antibodies (1:100 anti-Tuj1) overnight at 4°C. The next day, after three 10-min PBS washes, the samples were incubated with secondary antibody (1:500 dilution of Alexa Fluor 488 goat anti-rabbit IgG, Invitrogen Thermo Fisher Scientific Life Technologies, stock concentration 2 mg/mL) for an additional 1 h at room temperature, followed by three times of 10-min PBS washes. The samples were stored in PBS at 4°C. Images were captured using a Leica total internal reflection fluorescent microscope (TIRF & Thunder, DMi8S).

### Mitochondrial parameter analysis

Mitochondrial aspect ratios (long axis/short axis) and perimeters were calculated using the Mitochondrial Analyzer plugin in Image J (FIJI). Mitochondrial images were skeletonized, followed by 2D per-mito analysis.

### Mitochondrial axonal transport analysis

The series of mitochondrial transport images were imported into ImageJ (FiJi). Five axons were selected in each image series where mitochondrial transport could be visualized without interference from overlapping axons. Kymographs were generated using the “Kymograph” macro in FiJi. The velocities of individual mitochondria were analyzed using KymographDirect 2.1, based on the ratio of the individual mitochondrial travel distance to the total imaging time (300 s).

## Conflict of interest

Yang Liu, Chen Yan, Borui Cao, Dejun Kong, Jiaqi Li, Wenlei Li, Yingjie Guo, Zhongyang Yuan, Yumiao Gao, Yubo Zhang, Ran Sui, Guo Chen, Xiaojiang Hao and Quan Chen declare that they have no conflict of interest.

## References

[bBarreto2016] (2016). Epidemiologic study of Charcot-Marie-Tooth disease: a systematic review. Neuroepidemiology.

[bBellofatto2023] (2023). Anxiety and depression in Charcot-Marie-Tooth disease: data from the Italian CMT national registry. J Neurol.

[bBraathen2012] (2012). Genetic epidemiology of Charcot-Marie-Tooth disease. Acta Neurol Scand Suppl.

[bCao2017] (2017). MFN1 structures reveal nucleotide-triggered dimerization critical for mitochondrial fusion. Nature.

[bChen2003] (2003). Mitofusins Mfn1 and Mfn2 coordinately regulate mitochondrial fusion and are essential for embryonic development. J Cell Biol.

[bChen2020] (2020). Mfn2 is involved in intervertebral disc degeneration through autophagy modulation. Osteoarthritis Cartilage.

[bChevrollier2024] (2024). Homozygous MFN2 variants causing severe antenatal encephalopathy with clumped mitochondria. Brain.

[bChung2008] (2008). Early-onset stroke associated with a mutation in mitofusin 2. Neurology.

[bChung2006] (2006). Early onset severe and late-onset mild Charcot-Marie-Tooth disease with mitofusin 2 (MFN2) mutations. Brain.

[bCui2023] (2023). Diverse CMT2 neuropathies are linked to aberrant G3BP interactions in stress granules. Cell.

[bDetmer2007] (2007). Complementation between mouse Mfn1 and Mfn2 protects mitochondrial fusion defects caused by CMT2A disease mutations. J Cell Biol.

[bDorn2019] (2019). Evolving concepts of mitochondrial dynamics. Annu Rev Physiol.

[bFeely2011] (2011). MFN2 mutations cause severe phenotypes in most patients with CMT2A. Neurology.

[bFerre2024] Ferre M (2024) Global Variome shared Leiden Open Variation Database: Unique variants in the MFN2 gene. https://databases.lovd.nl/shared/variants/MFN2/unique

[bFranco2016] (2016). Correcting mitochondrial fusion by manipulating mitofusin conformations. Nature.

[bGiacomello2020] (2020). The cell biology of mitochondrial membrane dynamics. Nat Rev Mol Cell Biol.

[bGuo2023] (2023). Small molecule agonist of mitochondrial fusion repairs mitochondrial dysfunction. Nat Chem Biol.

[bHao1987] (1987). The structures of four new diterpene alkaloids, spiramines A, B, C, and D. Chem Pharm Bull.

[bHuang2017] (2017). Sequences flanking the transmembrane segments facilitate mitochondrial localization and membrane fusion by mitofusin. Proc Natl Acad Sci USA.

[bIshihara2004] (2004). Mitofusin 1 and 2 play distinct roles in mitochondrial fusion reactions via GTPase activity. J Cell Sci.

[bLegros2002] (2002). Mitochondrial fusion in human cells is efficient, requires the inner membrane potential, and is mediated by mitofusins. Mol Biol Cell.

[bLi2019] (2019). Structural insights of human mitofusin-2 into mitochondrial fusion and CMT2A onset. Nat Commun.

[bLiu2022] (2022). Multi-color live-cell STED nanoscopy of mitochondria with a gentle inner membrane stain. Proc Natl Acad Sci USA.

[bMcCray2021] (2021). Axonal Charcot-Marie-Tooth disease: from common pathogenic mechanisms to emerging treatment opportunities. Neurotherapeutics.

[bMisko2010] (2010). Mitofusin 2 is necessary for transport of axonal mitochondria and interacts with the Miro/Milton complex. J Neurosci.

[bNascimento2018] (2018). The intriguing nature of dorsal root ganglion neurons: Linking structure with polarity and function. Prog Neurobiol.

[bPipis2020] (2020). Natural history of Charcot-Marie-Tooth disease type 2A: a large international multicentre study. Brain.

[bSharma2021] Sharma G, Zaman M, Sabouny R, Joel M, Martens K, Martino D, de Koning APJ, Pfeffer G, Shutt TE (2021) Characterization of a novel variant in the HR1 domain of MFN2 in a patient with ataxia, optic atrophy and sensorineural hearing loss. F1000Res 10: 606-638

[bStrickland2014] (2014). Characterization of the mitofusin 2 R94W mutation in a knock-in mouse model. J Peripher Nerv Syst.

[bStuppia2015] (2015). MFN2-related neuropathies: clinical features, molecular pathogenesis and therapeutic perspectives. J Neurol Sci.

[bSwinney2011] Swinney DC, Anthony J (2011) How were new medicines discovered? Nat Rev Drug Discov 10(7): 507-519

[bVerhoeven2006] (2006). MFN2 mutation distribution and genotype/phenotype correlation in Charcot-Marie-Tooth type 2. Brain.

[bWestermann2010] (2010). Mitochondrial fusion and fission in cell life and death. Nat Rev Mol Cell Biol.

[bZhou2019] (2019). Restoring mitofusin balance prevents axonal degeneration in a Charcot-Marie-Tooth type 2A model. J Clin Invest.

[bZuchner2004] (2004). Mutations in the mitochondrial GTPase mitofusin 2 cause Charcot-Marie-Tooth neuropathy type 2A. Nat Genet.

